# Whole-Genome Sequencing of 117 Chromosome Segment Substitution Lines for Genetic Analyses of Complex Traits in Rice

**DOI:** 10.1186/s12284-022-00550-y

**Published:** 2022-01-13

**Authors:** Jiongjiong Fan, Hua Hua, Zhaowei Luo, Qi Zhang, Mengjiao Chen, Junyi Gong, Xin Wei, Zonghua Huang, Xuehui Huang, Qin Wang

**Affiliations:** 1 Shanghai Key Laboratory of Plant Molecular Sciences, College of Life Sciences, Shanghai Normal University, 100 Guilin Road, Shanghai, 200234 China; 2 State Key Laboratory of Rice Biology, China National Rice Research Institute, Chinese Academy of Agricultural Sciences, Hangzhou, 310006 China

**Keywords:** Rice, Chromosome segment substitution lines (CSSLs), QTL mapping, Heading date, RFT1

## Abstract

Rice is one of the most important food crops in Asia. Genetic analyses of complex traits and molecular breeding studies in rice greatly rely on the construction of various genetic populations. Chromosome segment substitution lines (CSSLs) serve as a powerful genetic population for quantitative trait locus (QTL) mapping in rice. Moreover, CSSLs containing target genomic regions can be used as improved varieties in rice breeding. In this study, we developed a set of CSSLs consisting of 117 lines derived from the recipient ‘Huanghuazhan’ (HHZ) and the donor ‘Basmati Surkb 89–15’ (BAS). The 117 lines were extensively genotyped by whole-genome resequencing, and a high-density genotype map was constructed for the CSSL population. The 117 CSSLs covered 99.78% of the BAS genome. Each line contained a single segment, and the average segment length was 6.02 Mb. Using the CSSL population, we investigated three agronomic traits in Shanghai and Hangzhou, China, and a total of 25 QTLs were detected in both environments. Among those QTLs, we found that *RFT1* was the causal gene for heading date variance between HHZ and BAS. *RFT1* from BAS was found to contain a loss-of-function allele based on yeast two-hybrid assay, and its causal variation was a P to S change in the 94th amino acid of the RFT1 protein. The combination of high-throughput genotyping and marker-assisted selection (MAS) is a highly efficient way to construct CSSLs in rice, and extensively genotyped CSSLs will be a powerful tool for the genetic mapping of agronomic traits and molecular breeding for target QTLs/genes.

**Supplementary Information:**

The online version contains supplementary material available at 10.1186/s12284-022-00550-y.

## Background

Rice is an important food crop, and its high and stable yield is related to global food security. Many agronomic traits in rice, such as heading date, tiller number, plant height and disease resistance, are related to rice production, and these complex traits are controlled by many QTLs (Glazier et al. 2002). Five QTLs for heading date, namely, *Hd1*, *Hd2*, *Hd3*, *Hd4* and *Hd5,* were found in an F_2_ population derived from a Nipponbare and Kasalath cross (Yano et al. 1997). Through fine mapping, the rice *Hd1* gene (homolog of CONSTANS in *Arabidopsis*) was finally cloned and validated (Yano et al. 2000). In addition to heading date, QTL mapping and cloning have also been used in traits controlling plant architecture and stress resistance. For example, the *PROG1* gene, which is related to tiller angle and the number of tillers of rice (Jin et al. 2008; Tan et al. 2008), and *Thermotolerance 1* (*TT1*) for thermotolerance (Li et al. 2015), have been mapped. Therefore, QTL mapping and gene cloning are accurate and effective methods to study functional genes (Price 2006). To date, more than 225 QTLs have been cloned and functionally validated in rice (Salvi and Tuberosa 2005).

The mapping populations commonly used for QTL mapping include F_2_, F_2:3_, recombinant inbred lines (RILs), doubled haploid (DH), CSSLs, and others. As a stable mapping population, CSSLs have been widely used in QTL mapping and gene cloning. After the first application of CSSLs in tomato (Eshed and Zamir 1994, 1995), this technique was immediately applied to rice research (Doi et al. 1997). In general, the development of CSSLs requires MAS to determine the genotype of the population and perform backcross breeding. Ideally, each CSSL has a single, small minimal chromosome fragment from the donor, and all donor fragments collectively cover the entire genome of the donor (Balakrishnan et al. 2019). However, to obtain a perfect set of CSSLs, high-density molecular markers are needed to identify the size of the introgressed fragment, but the PCR analysis of molecular markers often greatly increases the workload. High-throughput genotyping methods based on next-generation sequencing technology can be used to draw high-resolution physical maps quickly, which can replace marker-based genotyping approaches and save many hours of laborious work (Huang et al. 2009). Recently, many high-precision CSSLs have been constructed by using high-throughput genotyping technology (Zhang et al. 2019; Zhu et al. 2015; Xu et al. 2010; Jiang et al. 2017). These high-quality CSSLs are helpful for analyzing traits and cloning candidate genes.

Flowering is the hallmark of the transition from vegetative growth to reproductive growth (Arteca 1996). For rice, flowering time (called heading date in rice) is directly related to yield. For example, *Ghd7* (Xue et al. 2008), *Ghd7.1*/*DTH7*/*OsPRR37* (Yan et al. 2013; Gao et al. 2014; Koo et al. 2013) and *Ghd8*/*DTH8* (Yan et al. 2011; Wei et al. 2010) simultaneously control three traits – grain yield, plant height, and heading date. In particular, florigen, as a key protein encoded by the *FLOWERING LOCUS T* (*FT*) gene, is directly related to flowering in plants; it is produced in the phloem of leaves and transferred to the shoot apical meristem (SAM) to induce flowering (Tsuji 2017; Turck et al. 2008; Tamaki et al. 2007). In rice, *Hd3a* and *RFT1* are orthologs of the *A. thaliana* florigen *FT*, with high sequence similarity (Komiya et al. 2008; Kojima et al. 2002). Previous studies have found that the 14-3-3 protein of the florigen receptor mediates the interaction of Hd3a and the transcription factor OsFD1 to form a triple-structured "florigen activation complex (FAC)" that activates the expression of the downstream genes *OsMADS14* and *OsMADS15* to induce rice heading (Taoka et al. 2011; Tamaki et al. 2015). Interestingly, RFT1 also interacts with the 14-3-3 protein, and nonfunctional RFT1 with the E105K mutation fails to interact with the 14-3-3 protein (Zhao et al. 2015). However, it is unclear whether other mutated sites in RFT1 can affect its interaction with the 14-3-3 protein.

Here, we constructed a set of CSSLs derived from the *indica* cultivar ‘Huanghuazhan’ (HHZ, a high-quality rice variety widely cultivated in China) and ‘Basmati Surkb 89–15’ (BAS, an aromatic rice variety from Pakistan). The variety HHZ was used as the recipient parent, and BAS was used as the donor parent. A total of 117 CSSLs were constructed by a combination of MAS and high-throughput genotyping based on whole-genome sequencing. QTLs for heading date, plant height and panicle length were analyzed using the CSSLs, and the biological function of RFT1 in BAS, which contained a P94S mutation, was verified.

## Results

### Development of the CSSLs

**Fig. 1:**
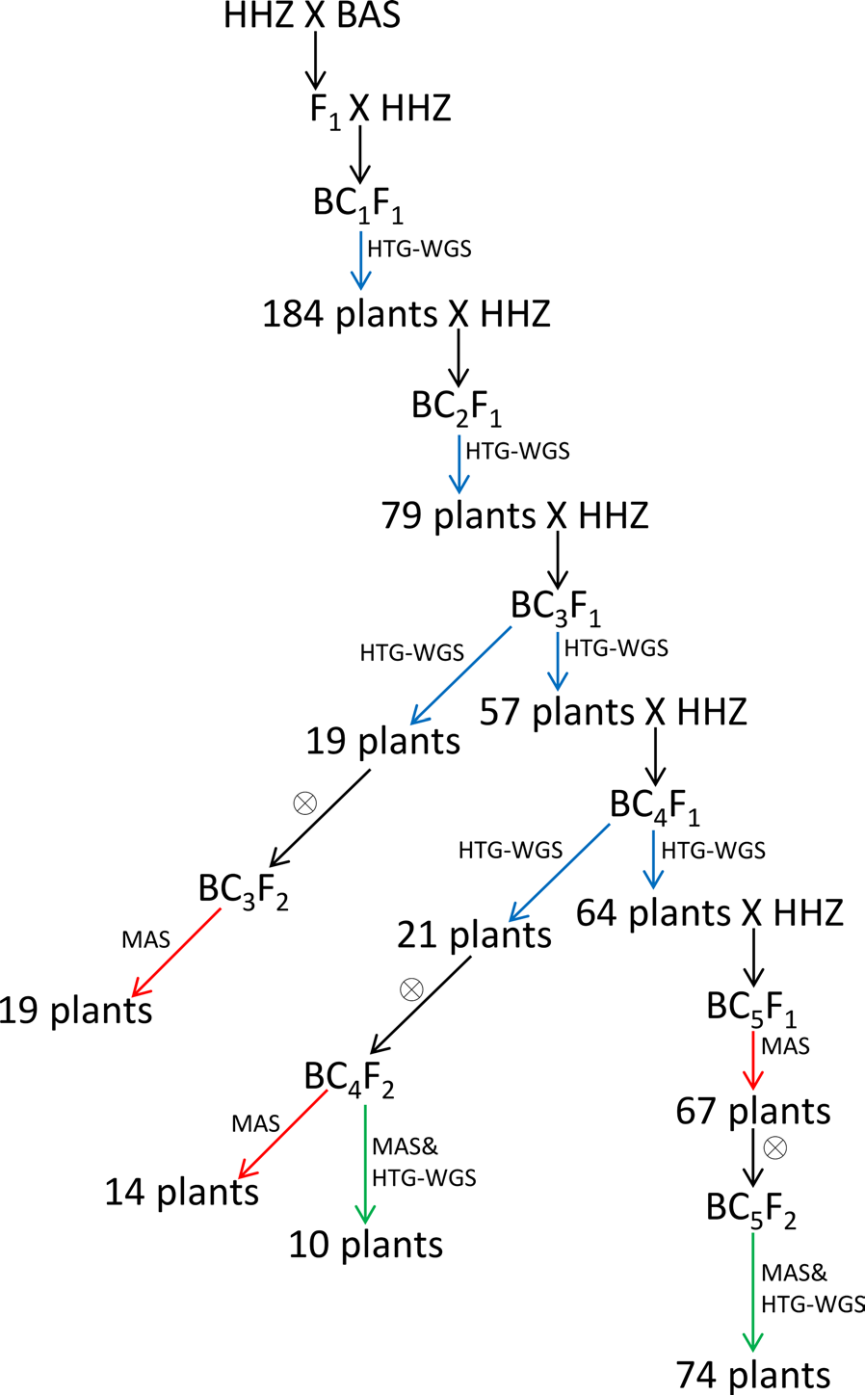
Flow charts of the 117 CSSLs constructed in this study. The red arrow represents lines genotyped by MAS. The blue arrow represents lines genotyped by HTG-WGS (High-Throughput Genotyping by Whole-Genome Resequencing). The green arrow represents lines genotyped by MAS and HTG-WGS. The black circle represents self-pollination

The development process of the CSSLs is shown in Fig. 1. F_1_ plants were obtained in cross between HHZ and BAS. The F_1_ plants were backcrossed once with HHZ to produce the BC_1_F_1_ generation. A total of 184 plants screened from the BC_1_F_1_ population were backcrossed to produce the BC_2_F_1_ generation. Then, 79 plants were backcrossed to produce the BC_3_F_1_ generation. Furthermore, 57 plants were screened from the BC_3_F_1_ population and backcrossed to create the BC_4_F_1_ generation, and 64 plants were screened from the BC_4_F_1_ population and backcrossed to create the BC_5_F_1_ generation. In each generation, plants that had heterozygous genotypes on one chromosome and the remaining genetic background homozygous for HHZ genotypes were chosen. In addition, heterozygous fragments of those selected plants could cover whole chromosomes. The genotypes of BC_n(1–4)_F_1_ plants were determined by whole-genome resequencing. The genotypes of BC_5_F_1_ plants were determined by MAS. A total of 107 plants, including 19 BC_3_F_1_ plants, 21 BC_4_F_1_ plants and 67 BC_5_F_1_ plants with a heterozygous substituted segment, were self-pollinated to produce BC_3_F_2_, BC_4_F_2_ and BC_5_F_2_ populations, respectively. Thirty-three plants with small segment substitutions (approximately 5 Mb), including 19 BC_3_F_2_ plants and 14 BC_4_F_2_ plants selected by MAS, were self-pollinated to obtain 33 CSSLs. Then, 7 BC_4_F_1_ plants and 67 BC_5_F_1_ plants were self-pollinated to obtain 84 CSSLs, and the 84 CSSLs were subjected to another round of high-throughput genotyping by whole-genome resequencing. Finally, a linkage map was constructed for the 117 CSSLs.

In addition to whole-genome sequencing, we also developed a set of PCR-based markers for genotyping and gene pyramiding in the future. Based on the comparison of the HHZ and BAS genome assemblies (data not shown), we developed 396 InDel (insertion-deletion) markers for the construction of CSSLs that were evenly distributed on the 12 rice chromosomes (Additional file 1: Fig. S1). The average interval between two adjacent markers on the physical map was 0.94 Mb (Additional file 2: Table S1). The primer sequence information for the markers used in this study is shown in Additional file 3: Table S2. Both PCR genotyping and whole-genome sequencing were applied to BC_5_F_1_ plants. The genotyping results from the 396 markers were consistent with those from whole-genome resequencing.

### Characteristics of the CSSLs

**Fig. 2:**
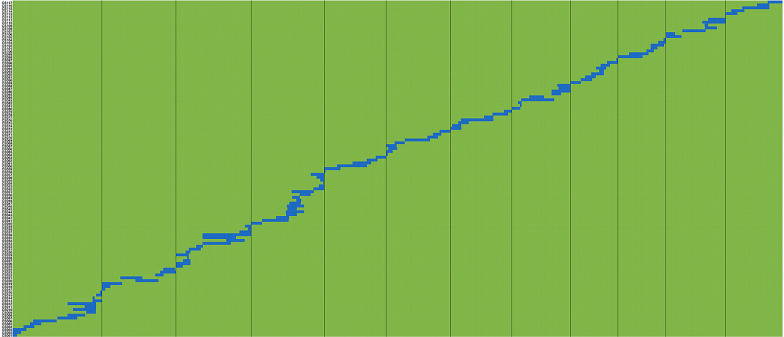
Bin-physical map of the 117 CSSLs constructed using molecular markers and whole-genome resequencing. Green and blue represent HHZ and BAS, respectively

**Fig. 3:**
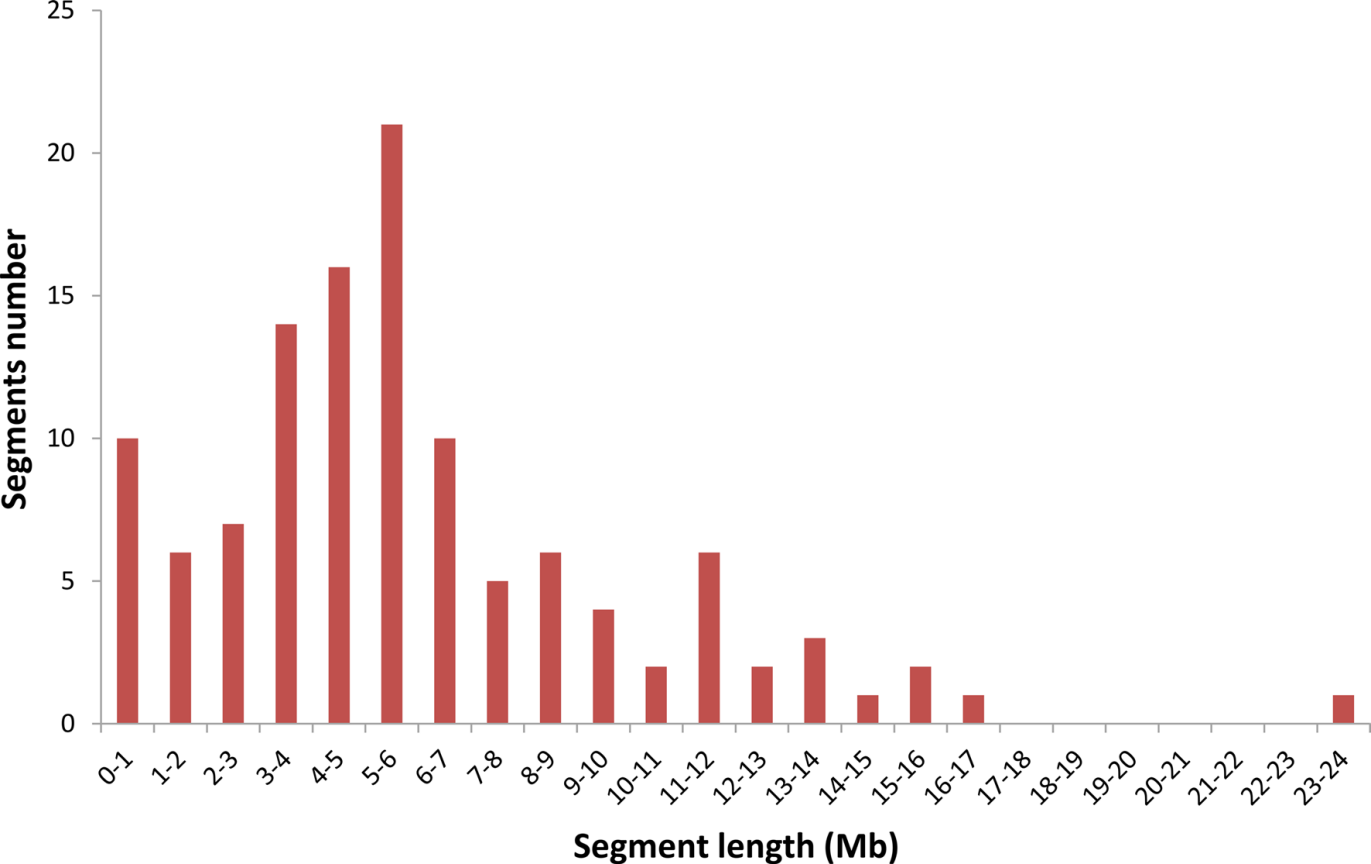
Distribution of the lengths of the substituted segments in the set of CSSLs

An accurate physical map of the 117 CSSLs was constructed according to SEG-Map (Zhao et al. 2010) with Os-Nipponbare-Reference-IRGSP-1.0 (Kawahara et al., 2013; Sakai et al. 2013) as the reference genome (Fig. 2). The set of CSSLs contains 117 homozygous segments, and each line contains only one substituted segment. The average number of substituted segments was approximately 10 for each chromosome, ranging from 5 on chromosome 12 to 18 on chromosome 4 (Table 1). Analysis of the length of the substituted segments showed that the total length of the substituted segments in the population was 704.6 Mb, which is 1.89 times the total length of the rice genome; on average, each line carried 6.02 Mb of substituted material. The coverage rate of the substituted segments with redundancy removed was 99.78% of the BAS genome in the CSSL set. Except for chromosome 11 and chromosome 1, which had 98.61% and 99.07% coverage rates, respectively, all of the other 10 chromosomes were fully covered. The size of the segments ranged from 0 to 24 Mb (Fig. 3). Among those segments, the smallest segment is 0.1 Mb, which is on chromosome 11, and the largest one is 23.5 Mb on chromosome 3. Additionally, 71.79% of the segments were shorter than 7 Mb, while 15.38% were longer than 10 Mb. In particular, 10 CSSLs had a substituted segment of less than 1 Mb (Fig. 3).

**Table 1:** Distribution of substituted segments on 12 chromosomes

Chromosome	Number of segments	Segment length (Mb)	Average length (Mb)	Coverage length (Mb)	Coverage rate (%)
1	16	96.1	6.00	42.8	99.07
2	8	54.1	6.76	35.8	100
3	15	103.7	6.91	36.3	100
4	18	98.6	5.48	35.4	100
5	6	41.6	6.93	29.9	100
6	9	44.2	4.91	31.1	100
7	7	45.5	6.50	29.6	100
8	9	55.0	6.11	28.3	100
9	9	38.9	4.32	22.9	100
10	7	38.8	5.54	23.1	100
11	8	50.2	6.28	28.0	98.61
12	5	37.9	7.58	27.4	100
Total	117	704.6	6.02	370.6	99.78

### QTL Analysis Using the CSSLs

**Fig. 4:**
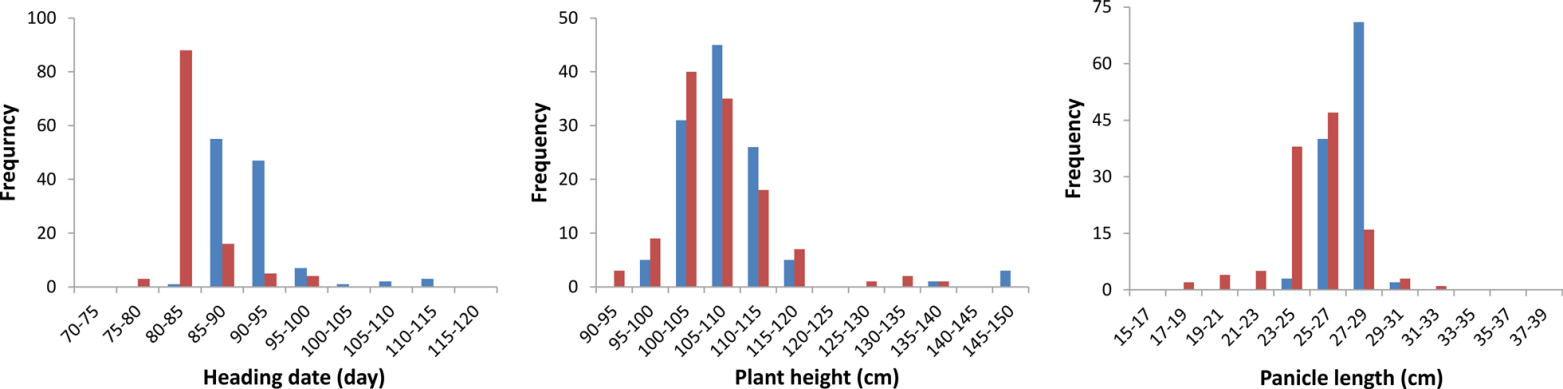
Frequency distributions of the three traits in the CSSLs. The blue and orange rectangles represent the distribution of the three traits in the CSSLs at the Shanghai and Hangzhou locations, respectively. The vertical axis of each figure represents the number of CSSL individuals

Since self-pollinated CS004 plants failed to produce seeds, heading time (HD), plant height (PH) and panicle length (PL) were investigated for 116 CSSLs and their parents in Shanghai and Hangzhou, China. The phenotypic values of the three traits had a normal or skewed distribution in both environments (Fig. 4). The average values for the CSSLs were close to the statistical data from HHZ, which was consistent with the genetic background of the CSSLs. Descriptive statistics are listed in Table 2. QTL IciMapping was used to analyze the QTLs for the specified agronomic traits in both Shanghai and Hangzhou (Table 3). A total of 25 QTLs were detected for those three traits and were distributed on 9 chromosomes, while no QTLs were found on chromosomes 2, 5, and 9 (Additional file 4: Fig. S2). Among the 25 QTLs, 9 were detected from the data derived from Shanghai, and 16 were detected from statistical data from Hangzhou; 4 significant QTLs (*qHD6-1*, *qHD8-1*, *qHD10-1*, and *qPH1-1*) were identified at both sites. Some QTLs were located in the same or adjacent chromosomal regions.

**Table 2:** Statistics of the three studied traits in BAS, HHZ and 116 CSSLs at the Shanghai and Hangzhou locations

Environment	Trait	BAS	HHZ	CSSLs			
				Mean	SD	CV(%)	Range
Shanghai	HD	123.4	91.7	91.5	4.9	5.3	84.6–114.7
	PH	185.5	107.0	108.7	8.2	7.6	97.9–149.1
	PL	30.2	27.2	27.3	1.0	3.5	24.2–29.3
Hangzhou	HD	103.0	83.0	83.9	3.8	4.5	75.0–100.0
	PH	190.0	107.0	107.1	7.3	6.8	93.5–136.5
	PL	32.0	26.5	25.5	2.3	8.7	18.5–32.0

*SD* standard deviation; *CV* coefficient of variation

**Table 3:** QTLs for the three traits detected in the set of CSSLs in Shanghai and Hangzhou

Environment	Trait	QTL	Chromosome	Location (bin)	Location (Mb)	LOD	PVE (%)^a^	Add^b^	Known QTL/gene
Shanghai	HD	*qHD1-1*	chr01	chr01_357	35.7–40.2	3.2	1.7	− 1.7	*OsMADS51*
Shanghai	HD	*qHD4-2*	chr04	chr04_196	18.5–22.0	14.1	9.3	3.4	
Shanghai	HD	*qHD6-1*	chr06	chr06_10	1.0–3.0	41.7	52.3	11.2	*Hd3a*/*RFT1*
Shanghai	HD	*qHD8-1*	chr08	chr08_41	3.9–4.6	22.9	18.4	8.1	*Ghd8*
Shanghai	HD	*qHD10-1*	chr10	chr10_159	15.9–17.3	14.5	9.7	4.8	*Ehd1*
Shanghai	PH	*qPH1-1*	chr01	chr01_357	35.7–40.2	34.5	71.7	19.2	*sd1*
Shanghai	PH	*qPH6-1*	chr06	chr06_10	1.0–3.0	3.5	3.6	5.0	*Hd3a*/*RFT1*
Shanghai	PL	*qPL3-2*	chr03	chr03_243	24.3–26.5	3.8	12.5	− 0.9	
Shanghai	PL	*qPL10-1*	chr10	chr10_174	15.9–19.0	3.0	9.8	− 1.2	
Hangzhou	HD	*qHD4-1*	chr04	chr04_185	18.5–22.0	9.6	3.8	1.8	
Hangzhou	HD	*qHD6-1*	chr06	chr06_10	1.0–3.0	49.7	50.5	8.5	*Hd3a*/*RFT1*
Hangzhou	HD	*qHD7-1*	chr07	chr07_0	0.0–5.1	10.9	4.4	4.3	
Hangzhou	HD	*qHD7-2*	chr07	chr07_207	20.3–27.7	5.2	1.9	2.8	
Hangzhou	HD	*qHD8-1*	chr08	chr08_41	3.9–4.6	18.4	8.8	4.3	*Ghd8*
Hangzhou	HD	*qHD10-1*	chr10	chr10_159	15.9–17.3	15.5	7.0	3.2	*Ehd1*
Hangzhou	HD	*qHD10-2*	chr10	chr10_195	19.5–22.3	5.2	1.9	2.0	
Hangzhou	HD	*qHD11-1*	chr11	chr11_193	18.9–20.4	6.5	2.4	1.9	
Hangzhou	HD	*qHD11-2*	chr11	chr11_288	28.8–28.9	6.5	2.4	1.9	
Hangzhou	HD	*qHD12-1*	chr12	chr12_57	2.7–9.0	5.2	1.9	2.8	
Hangzhou	HD	*qHD12-2*	chr12	chr12_91	8.0–20.4	15.8	7.2	− 5.5	
Hangzhou	HD	*qHD12-3*	chr12	chr12_204	20.4–21.1	43.4	37.5	7.3	
Hangzhou	HD	*qHD12-4*	chr12	chr12_212	21.1–27.4	11.6	4.8	− 4.5	
Hangzhou	PH	*qPH1-1*	chr01	chr01_357	35.7–40.2	17.4	46.7	13.8	*sd1*
Hangzhou	PH	*qPH8-1*	chr08	chr08_238	22.6–28.3	3.1	6.2	5.7	
Hangzhou	PL	*qPL3-1*	chr03	chr03_130	12.8–26.5	3.9	14.0	− 2.7	

^a^The percentage of phenotypic variation explained (PVE) by the detected QTL; ^b^ Additive effects (Add), the positive value suggests that alleles from BAS increase the effect

### Heading Date

**Fig. 5:**
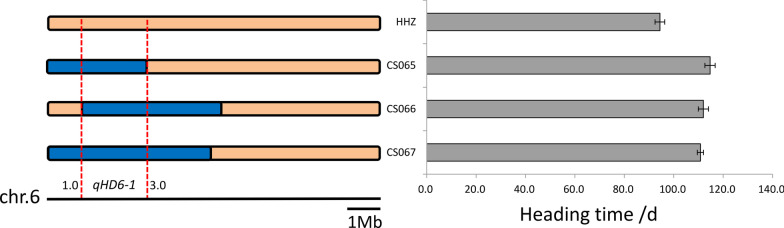
The genomic interval found in the progeny homozygous for *qHD6-1* delimited the locus to an ~ 2.0 Mb region. Different colors represent different genotypes: orange, HHZ; blue, BAS. Red dashed lines indicate the overlapping regions in different CSSLs. Heading date is shown for recombinant plants (CS065, CS066 and CS067) and the parental plants. Heading date values (in days) are shown as the means ± standard errors (n = 30)

Under long-day (LD) conditions, 15 QTLs associated with HD were detected on chromosomes 1, 4, 6, 7, 8, 10, 11 and 12. Among them, 3 QTLs (*qHD6-1*, *qHD8-1*, and *qHD10-1*) could be detected simultaneously in both planting environments. The phenotypic variation explained by individual QTLs was between 1.7 and 52.3%. For 12 QTLs (*qHD4-1*, *qHD4-2*, *qHD6-1*, *qHD7-1*, *qHD7-*2, *qHD8-1*, *qHD10-1*, *qHD10-2*, *qHD11-1*, *qHD11-2*, *qHD12-1*, and *qHD12-3*), the BAS alleles resulted in delayed heading, and for 3 QTLs (*qHD1-1*, *qHD12-2*, *qHD12-4*), the BAS alleles promoted heading. Among these QTLs, *qHD6-1* had the greatest impact on heading date and was located in the region from 1.0 to 3.0 Mb on chromosome 6 (Fig. 5), which contained two rice *FT* genes. Another site affecting the heading date, *qHD8-1*, was located between 3.9 and 4.6 Mb on chromosome 8, and *Ghd8* was supposed to be the causative gene. In addition, in the BC_3_F_1_ population created during the CSSLs construction process, four QTLs (*qHD1-1*, *qHD6-1*, *qHD8-1*, *qHD10-1*) were also detected when cultivated in Shanghai in 2019 (Additional file 5: Fig. S3).

### Plant Height

Three QTLs associated with PH were detected on chromosomes 1, 6 and 8. The phenotypic variance explained by individual QTLs varied from 3.6% to 71.7%. The QTL *qPH1-1*, for which the BAS genotype delayed heading date, explained 71.7% of the phenotypical variation in Shanghai and was located in the region from 35.7 to 40.2 Mb on chromosome 1. This region contained the rice green revolution gene *sd1*. BAS alleles at *qPH6-1* had positive effects on plant height in Shanghai, and this QTL was located at the same position as *qHD6-1*.

### Panicle Length

Three QTLs associated with PL were detected on chromosomes 3 and 10. The phenotypic variance explained by individual QTLs varied from 9.8% to 14.0%. The BAS alleles for three QTLs had negative effects on panicle length. However, no shared QTLs were detected in the two sites.

### Verification of the Biological Function of *qHD6-1*

QTL analysis was carried out for heading date in this set of CSSLs, and *qHD6-1* found on chromosome 6 had a noticeable effect on heading date. The interval of *qHD6-1* could be narrowed down to a region of 2.0 Mb, which was located from 1.0 Mb to 3.0 Mb on chromosome 6 (Fig. 5). Considering that *qHD6-1* is a major locus underlying heading date, we analyzed the candidate genes related to heading date in this region and preliminarily identified rice *FT* genes (*Hd3a* and *RFT1*) that may play a role in this locus.

**Fig. 6:**
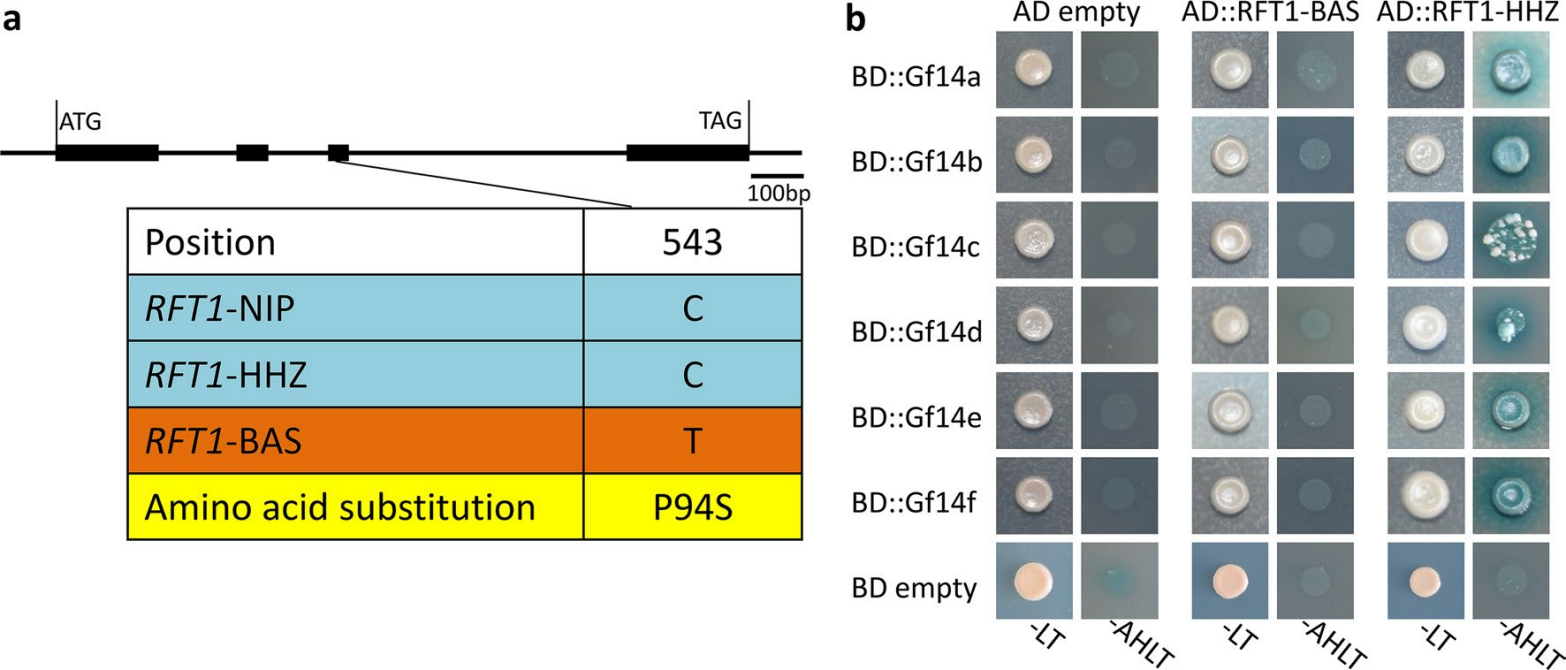
**a** The nucleotide sequences and amino acid sequence variation sites of RFT1 in HHZ and BAS compared with Nipponbare. **b** The protein interactions were tested by yeast two-hybrid assays. RFT1-HHZ interacted with six members of the 14-3-3 protein family, but RFT1-BAS did not interact with any. The interactions are indicated by blue-colored yeast colonies on SD/ − Ade/ − His/ − Leu/ − Trp/ + X-α-Gal/ + aureobasidin A (-AHLT) media. SD/-Leu/-Trp (-LT)

Sequence variation analysis of the coding region and 5 kb promoter of *Hd3a* and *RFT1* showed that there was a 1 bp deletion in the *Hd3a* promoter of BAS (Additional file 6: Fig. S4), and in *RFT1,* there was a nonsynonymous mutation in exon 3 (Fig. 6a). Compared with HHZ, RFT1-BAS has a unique amino acid substitution from Pro (P) to Ser (S) at position 94. Then, we analyzed the transcriptome data of 30-day-old seedling leaves and found that there was no difference in the expression of *Hd3a* between the two parents (data not shown). In recent studies, we collected quantitative trait gene (QTG) alleles of known QTLs and confirmed that the BAS alleles for *Hd3a* did not belong to the known QTG alleles (Wei et al. 2021). According to previous studies, the *Hd3a* promoter types of BAS and HHZ did not cause differences in the expression of *Hd3a* (Takahashi et al. 2009). In addition, *RFT1* played a major role in inducing rice flowering under LD conditions. Under these conditions, the heading date of *RFT1* RNAi plants was delayed by approximately 100 days compared with that of the wild type, whereas *Hd3a* RNAi plants basically flowered at the same time as wild-type plants (Komiya et al. 2009). Recently, *hd3a* and *rft1* were targeted by CRISPR/Cas9-mediated mutagenesis of *Hd3a* and *RFT1*. Under LD conditions, the heading date of *rft1* mutants was significantly delayed compared with that of wild-type plants (Liu et al. 2019; Song et al. 2017), while *hd3a* mutants did not display late-flowering phenotypes under those conditions (Song et al. 2017). In summary, we speculated that the candidate gene at *qHD6.1* was possibly *RFT1* rather than *Hd3a*.

To verify the functionality of RFT1-BAS, we performed yeast two-hybrid assays to test the interaction between the proteins encoded by the different *RFT1* alleles (HHZ and BAS) and 14-3-3 family proteins (Gf14a, Gf14b, Gf14c, Gf14d, Gf14e, and Gf14f). We found that RFT1-HHZ could interact with all the isoforms of GF14, while none of the GF14s interacted with RFT1-BAS (Fig. 6b). This result suggested that the P94S mutation in RFT1-BAS prevented the interaction with the 14-3-3 protein. Therefore, we proposed that *rft1*-BAS is a nonfunctional allele caused by a coding SNP that leads to the P94S substitution.

## Discussion

CSSLs are an excellent population for QTL mapping and gene cloning. Currently, a number of CSSLs populations with *indica* and *japonica* as parents have been successfully constructed (Kubo et al. 2002; Xi et al. 2006; Zhu et al. 2009). In addition, some groups have constructed CSSLs using cultivated rice as recurrent parents and wild rice as donors (Ma et al. 2019; Yuan et al. 2020; Xi et al. 2006). Based on the several CSSLs populations, many important functional genes have been cloned. A total of 153 single segment substitution lines (SSSLs) were constructed by crossing Basmati385 (donor parent) and HJX74 (recurrent parent), and *OsSPL16*, which controls grain size, grain shape and rice quality, was cloned from this population (Wang et al. 2012a; Zhang et al. 2004; Xi et al. 2006). *PROG1*, which controls the prostrate growth habit of common wild rice, was originally mapped from a set of CSSLs derived from Teqing as the recurrent parent and wild rice (*O. rufipogon*) as the donor parent (Hao et al. 2006; Jin et al. 2008). In this study, we mapped a number of novel QTLs using 116 CSSLs that will be used for gene cloning in future studies. For example, *qHD4-2* for heading date was mapped between 18.5 and 22.0 Mb on chromosome 4; genes related to heading date have not been reported in this region. Therefore, this set of CSSLs provides excellent material for QTL mapping and cloning.

CSSLs contain one substituted chromosomal segment from the donor parent, so they can be used as near-isogenic lines (NILs) by themselves or can be developed into higher resolution NILs by crossing with the recurrent parent again (Zamir 2001). NILs must be constructed when cloning genes using the traditional QTL cloning method (Zhang et al. 2006). To genotype a CSSLs population, molecular markers that are inexpensive and easy to use must be adopted. Currently, multifarious molecular marker systems have been established. However, in the process of constructing CSSLs by the MAS method, the size of the substituted fragment cannot be accurately calculated; therefore, deviations may occur in QTL detection (Paterson et al. 1990). High-throughput genotyping by whole-genome resequencing can accurately determine recombination breakpoints (Huang et al. 2009), which have been used for physical mapping of RIL, F_2_ and CSSL populations (Wang et al. 2011; Huang et al. 2016; Xu et al. 2010).

The stable production of rice is directly related to global food security. Therefore, breeding varieties with high yield, strong stress resistance (biotic and abiotic stresses) and superior quality should be a top priority for breeders (Zhang 2007). However, using traditional breeding methods to improve multiple crop traits simultaneously is difficult (Schaart et al. 2016). Recently, the concept of rational design breeding was proposed, and valuable genes from different rice varieties were pyramided to simultaneously improve multiple traits in Teqing in a short time (Zeng et al. 2017). In previous studies, according to RiceNavi, we pyramided *Badh2* (Chen et al. 2008), *TAC1* (Yu et al. 2007) and *OsSOC1* (Lee et al. 2004) from BAS, and the new line showed improved grain fragrance, heading date, plant type and yield compared with HHZ (Wei et al. 2021). The 117 CSSLs created here can be applied to the innovation of germplasm resources. Moreover, we also designed 396 InDel markers based on HHZ and BAS genome sequences; these markers can be applied to gene pyramiding of different chromosome segments.

Even though each CSSL contains only one segment, the QTL mapping interval is still large. However, quantitative trait nucleotide (QTN) variation information of some major loci in rice has been extensively studied (Wei et al. 2021), and QTNs can be located in many different populations. Therefore, even if some QTLs are located in a large interval, the main causal genes contained in this interval can be analyzed based on previous research results and existing biological techniques. For instance, the BAS allele at locus *qPH1-1*, which can increase plant height, was located at 35.7–40.2 Mb on chromosome 1, where *SD1*-BAS was confirmed as a wild-type allele. Furthermore, rice *FT* is candidate gene of *qHD6-1*. By combining different analysis and experimental methods, we confirmed with high probability that the candidate gene of *qHD6-1* is *RFT1*.

## Conclusions

In summary, we successfully developed a set of CSSLs by combining MAS and high-throughput genotyping based on whole-genome resequencing. These CSSLs can be used for QTL mapping, cloning and molecular breeding. Using this set of CSSLs, we not only detected cloned QTLs but also found some novel QTLs. Among them, a new nonfunctional *rft1*-BAS allele was verified based on yeast two-hybrid experiments.

## Materials and Methods

### Plant Materials

Huanghuazhan (HHZ), an *indica* cultivar, is often used as a restorer line in three-line hybrid rice seed production. The aromatic rice Basmati Surkb 89–15 (BAS) is native to Pakistan. The variety HHZ was used as the recipient parent, and BAS was used as the donor parent. The two parental lines were derived from the China National Rice Research Institute. All materials used in the process of population construction were grown in the summer in Shanghai (121°42′ E, 30°97′ N) and in the winter in Sanya (109°19′ E, 18°38′ N), China.

### DNA Extraction and Molecular Analysis

The TPS method was used for the extraction of genomic DNA from fresh leaves of each individual. DNA amplification was performed by PCR with the following protocol: predenaturation at 94 °C for 5 min; 36 cycles of denaturation at 94 °C for 30 s, annealing at 53–58 °C for 30 s, and extension at 72 °C for 30 s; and a final extension at 72 °C for 5 min. The reactions were carried out in 96-well PCR plates in 25 μl volumes containing 50–100 ng of template DNA, 0.2 μmol/L of each primer and 12.5 μl of 2 × EasyTaq PCR SuperMix (TransGen Biotech Inc., China). Electrophoresis of the amplification products was carried out on 4% agarose gels and photographed using a Tanon 1600 Automatic Digital Gel Imaging Analysis System (Tanon Inc., China).

### High-Throughput Genotyping by Whole-Genome Resequencing

The genomic DNA from individuals from each generation used for sequencing was extracted from young leaves using magnetic beads (no. 500 T, NanoMagBio S-96, China). The Tn5 transposition system was used for DNA library construction. DNA libraries were sequenced with Illumina HiSeq X Ten or NovaSeq6000 using PE150 flow cells according to standard procedures and generated 150 bp paired-end reads with an average 500 bp insert size for subsequent genotyping analyses. Approximately 0.4 × coverage sequence reads were generated for each line. Genotyping was performed using SEG-Map software.

### Field Experimental Design and Phenotypic Assessment for CSSLs

The two parents and 116 CSSLs were planted in Shanghai (121°42′ E, 30°97′ N) and Hangzhou (119°93′ E, 30°08′ N) in the summer of 2021. Thirty-day-old seedlings of each line were transplanted into a seven-row plot with seven plants per row and 25 × 30 cm spacing. Field management followed local regulations. The middle five plants in each row were used as samples for phenotypic measurement.

Heading date was defined as the time from sowing to emergence of the first inflorescences above the flag leaf sheath. Plant height, panicle length, and tiller number were measured 20 days after heading. The distance from the ground to the top of the first panicle was measured as the full height of the plant. Panicle length was measured as the plant height minus the distance from the ground to the neck-panicle node. Thirty replications were performed for each trait in Shanghai, and two replications were performed in Hangzhou.

### QTL Analysis for Three Agronomic Traits Based on CSSLs

Based on the physical map, each line was converted into a skeleton bin map with 3723 bins. Using a 116 bin map and phenotypic data, QTL analysis was performed with QTL IciMapping V4.2.53 software (https://www.isbreeding.net/). WinQTLCart (Wang et al. 2012b) was used to analyze the heading time in the BC_3_F_1_ population, and the relevant parameters were set according to the user manual.

Using CSSLs with overlapping substituted fragments, the location of QTLs can be predicted by substitution mapping (Paterson et al. 1990). MapChart2.32 (https://www.wur.nl/en/show/MapChart-2.32.htm) was used to map the distribution of QTLs on chromosomes.

### RNA Extraction and Yeast Two-Hybrid (Y2H) Assay

Total RNA was extracted using TRIzol reagent (Invitrogen Inc.) following the manufacturer’s instructions. First-strand cDNA was retrotranscribed using reverse transcriptase (Takara Bio Inc.). The vectors and yeast strains used in the yeast two-hybrid assays were from Clontech (Beijing, China).

When studying the interaction between the RFT1-HHZ/BAS and 14-3-3 family proteins, the coding sequences of *RFT1* (BAS; HHZ: *LOC_Os06g06300*) were cloned into pGADT7. The 14-3-3 gene sequences (*GF14a*: *Os08g0480800*; *GF14b*: *Os04g0462500*; *GF14c*: *Os08g0430500*; *GF14d*: *Os11g0546900*; *GF14e*: *Os02g0580300*; and *GF14f*: *Os03g0710800*) were cloned into pGBKT7. The vectors were cotransformed with AD-RFT1s, and the transformed cells were selected on SD-Ade/-His/-Trp/-Leu/ + X-α-Gal/ + aureobasidin A medium. Assays were performed according to the Yeast Protocols Handbook (Clontech). Primers for the yeast two-hybrid vectors are listed in Additional file 7: Table S3.

## Supplementary Information

**Additional file 1.** **Fig. S1** Location of 396 InDel marker primers on chromosomes; the black bar represents a marker.

**Additional file 2.** **Table S1** Distribution of InDel markers on 12 rice chromosomes used to construct the set of CSSLs.

**Additional file 3.** **Table S2** Primer pairs of 396 InDel markers.

**Additional file 4.** **Fig. S2** QTL distribution on chromosomes. The blue font denotes loci found in both environments, the red font denotes loci that can only be found in Shanghai, and the green font denotes loci that can only be found in Hangzhou.

**Additional file 5.** **Fig. S3** Mapping of heading date in the BC_3_F_1_ population by WinQTLCart.

**Additional file 6.** **Fig. S4** Nucleotide polymorphism of the *Hd3a *promoter between HHZ and BAS.

**Additional file 7.** **Table S3** Primers for yeast two-hybrid vectors.

## Data Availability

All data generated or analyzed during this study are included in this published article and its supplementary information files. The CSSL materials are available from the corresponding author.

## Abbreviations

BAS: Basmati Surkb 89–15;
CSSLs: Chromosome segment substitution lines
DH: Doubled haploid
HD: Heading date
InDel: Insertions and deletions
HHZ: Huanghuazhan
LOD: Logarithm of odds
LD: Long-day
MAS: Marker-assisted selection
NILs: Near-isogenic lines
PCR: Polymerase chain reaction
PH: Plant height
PL: Panicle length
QTG: Quantitative trait gene
QTL: Quantitative trait locus
QTN: Quantitative trait nucleotide
RILs: Recombinant inbred lines
SAM: Shoot apical meristem
SSSLs: Single segment substitution lines
Y2H: Yeast two-hybrid

## References

[CR1] Arteca RN (1996) Flowering. In: Plant growth substances. Springer, Boston, MA. pp 177–187 10.1007/978-1-4757-2451-6_8.

[CR2] Balakrishnan D, Surapaneni M, Mesapogu S, Neelamraju S (2019). Development and use of chromosome segment substitution lines as a genetic resource for crop improvement. Theor Appl Genet.

[CR3] Chen S, Yang Y, Shi W, Ji Q, He F, Zhang Z, Cheng Z, Liu X, Xu M (2008). *Badh2*, encoding betaine aldehyde dehydrogenase, inhibits the biosynthesis of 2-acetyl-1-pyrroline, a major component in rice fragrance. Plant Cell.

[CR4] Doi K, Iwata N, Yoshimura A (1997). The construction of chromosome substitution lines of African rice (Oryza glaberrima Steud.) in the background of Japonica rice (O. sativa L.). Rice Genet Newslett.

[CR5] Eshed Y, Zamir D (1994). A genomic library of Lycopersicon pennellii in L. esculentum: A tool for fine mapping of genes. Euphytica.

[CR6] Eshed Y, Zamir D (1995). An introgression line population of *Lycopersicon pennellii* in the cultivated tomato enables the identification and fine mapping of yield-associated QTL. Genetics.

[CR7] Gao H, Jin M, Zheng XM, Chen J, Yuan D, Xin Y, Wang M, Huang D, Zhang Z, Zhou K, Sheng P, Ma J, Ma W, Deng H, Jiang L, Liu S, Wang H, Wu C, Yuan L, Wan J (2014). *Days to heading 7*, a major quantitative locus determining photoperiod sensitivity and regional adaptation in rice. Proc Natl Acad Sci USA.

[CR8] Glazier AM, Nadeau JH, Aitman TJ (2002). Finding genes that underlie complex traits. Science.

[CR9] Hao W, Jin J, Sun SY, Zhu MZ, Lin HX (2006). Construction of chromosome segment substitution lines carrying overlapping chromosome segments of the whole wild rice genome and identification of quantitative trait loci for rice quality. J Plant Physiol Mol Biol.

[CR10] Huang X, Feng Q, Qian Q, Zhao Q, Wang L, Wang A, Guan J, Fan D, Weng Q, Huang T, Dong G, Sang T, Han B (2009). High-throughput genotyping by whole-genome resequencing. Genome Res.

[CR11] Huang X, Yang S, Gong J, Zhao Q, Feng Q, Zhan Q, Zhao Y, Li W, Cheng B, Xia J, Chen N, Huang T, Zhang L, Fan D, Chen J, Zhou C, Lu Y, Weng Q, Han B (2016). Genomic architecture of heterosis for yield traits in rice. Nature.

[CR12] Jiang N, Shi S, Shi H, Khanzada H, Wassan GM, Zhu C, Peng X, Yu Q, Chen X, He X, Fu J, Hu L, Xu J, Ouyang L, Sun X, Zhou D, He H, Bian J (2017). Mapping QTL for seed germinability under low temperature using a new high-density genetic map of rice. Front Plant Sci.

[CR13] Jin J, Huang W, Gao JP, Yang J, Shi M, Zhu MZ, Luo D, Lin HX (2008). Genetic control of rice plant architecture under domestication. Nat Genet.

[CR14] Kawahara Y, de la Bastide M, Hamilton JP, Kanamori H, McCombie WR, Ouyang S (2013). Improvement of the Oryza sativa Nipponbare reference genome using next generation sequence and optical map data. Rice..

[CR15] Kojima S, Takahashi Y, Kobayashi Y, Monna L, Sasaki T, Araki T, Yano M (2002). *Hd3a*, a rice ortholog of the *Arabidopsis FT* gene, promotes transition to flowering downstream of *Hd1* under short-day conditions. Plant Cell Physiol.

[CR16] Komiya R, Ikegami A, Tamaki S, Yokoi S, Shimamoto K (2008). *Hd3a* and *RFT1* are essential for flowering in rice. Development.

[CR17] Komiya R, Yokoi S, Shimamoto K (2009). A gene network for long-day flowering activates *RFT1* encoding a mobile flowering signal in rice. Development.

[CR18] Koo BH, Yoo SC, Park JW, Kwon CT, Lee BD, An G, Zhang Z, Li J, Li Z, Paek NC (2013). Natural variation in *OsPRR37* regulates heading date and contributes to rice cultivation at a wide range of latitudes. Mol Plant.

[CR19] Kubo T, Aida Y, Nakamura K, Tsunematsu H, Doi K, Yoshimura A (2002). Reciprocal chromosome segment substitution series derived from Japonica and Indica cross of rice (Oryza sativa L.). Breeding Sci.

[CR20] Lee S, Kim J, Han JJ, Han MJ, An G (2004). Functional analyses of the flowering time gene *OsMADS50*, the putative *SUPPRESSOR OF OVEREXPRESSION OF CO 1*/*AGAMOUS-LIKE 20* (*SOC1*/*AGL20*) ortholog in rice. Plant J.

[CR21] Li XM, Chao DY, Wu Y, Huang X, Chen K, Cui LG, Su L, Ye WW, Chen H, Chen HC, Dong NQ, Guo T, Shi M, Feng Q, Zhang P, Han B, Shan JX, Gao JP, Lin HX (2015). Natural alleles of a proteasome α2 subunit gene contribute to thermotolerance and adaptation of African rice. Nat Genet.

[CR22] Liu B, Liu Y, Wang B, Luo Q, Shi J, Gan J, Shen WH, Yu Y, Dong A (2019). The transcription factor OsSUF4 interacts with SDG725 in promoting H3K36me3 establishment. Nat Commun.

[CR23] Ma X, Han B, Tang J, Zhang J, Cui D, Geng L, Zhou H, Li M, Han L (2019). Construction of chromosome segment substitution lines of Dongxiang common wild rice (*Oryza rufipogon* Griff.) in the background of the *japonica* rice cultivar Nipponbare (*Oryza sativa* L.). Plant Physiol Biochem.

[CR24] Paterson AH, DeVerna JW, Lanini B, Tanksley SD (1990). Fine mapping of quantitative trait loci using selected overlapping recombinant chromosomes, in an interspecies cross of tomato. Genetics.

[CR25] Price AH (2006). Believe it or not, QTLs are accurate!. Trends Plant Sci.

[CR26] Sakai H, Lee SS, Tanaka T, Numa H, Kim J, Kawahara Y (2013). Rice annotation project database (RAP-DB): an integrative and interactive database for rice genomics. Plant Cell Physiol.

[CR27] Salvi S, Tuberosa R (2005). To clone or not to clone plant QTLs: present and future challenges. Trends Plant Sci.

[CR28] Schaart JG, van de Wiel CCM, Lotz LAP, Smulders MJM (2016). Opportunities for products of new plant breeding techniques. Trends Plant Sci.

[CR29] Song S, Chen Y, Liu L, Wang Y, Bao S, Zhou X, Teo ZW, Mao C, Gan Y, Yu H (2017). OsFTIP1-Mediated regulation of Florigen transport in rice is negatively regulated by the ubiquitin-like domain kinase OsUbDKγ4. Plant Cell.

[CR30] Takahashi Y, Teshima KM, Yokoi S, Innan H, Shimamoto K (2009). Variations in Hd1 proteins, *Hd3a* promoters, and *Ehd1* expression levels contribute to diversity of flowering time in cultivated rice. Proc Natl Acad Sci.

[CR31] Tamaki S, Matsuo S, Wong HL, Yokoi S, Shimamoto K (2007). Hd3a protein is a mobile flowering signal in rice. Science.

[CR32] Tamaki S, Tsuji H, Matsumoto A, Fujita A, Shimatani Z, Terada R, Sakamoto T, Kurata T, Shimamoto K (2015). FT-like proteins induce transposon silencing in the shoot apex during floral induction in rice. Proc Natl Acad Sci USA.

[CR33] Tan L, Li X, Liu F, Sun X, Li C, Zhu Z, Fu Y, Cai H, Wang X, Xie D, Sun C (2008). Control of a key transition from prostrate to erect growth in rice domestication. Nat Genet.

[CR34] Taoka K, Ohki I, Tsuji H, Furuita K, Hayashi K, Yanase T, Yamaguchi M, Nakashima C, Purwestri YA, Tamaki S, Ogaki Y, Shimada C, Nakagawa A, Kojima C, Shimamoto K (2011). 14–3-3 proteins act as intracellular receptors for rice Hd3a florigen. Nature.

[CR35] Tsuji H (2017). Molecular function of florigen. Breed Sci.

[CR36] Turck F, Fornara F, Coupland G (2008). Regulation and identity of florigen: flowering locus T moves center stage. Annu Rev Plant Biol.

[CR37] Wang L, Wang A, Huang X, Zhao Q, Dong G, Qian Q, Sang T, Han B (2011). Mapping 49 quantitative trait loci at high resolution through sequencing-based genotyping of rice recombinant inbred lines. Theor Appl Genet.

[CR38] Wang S, Basten C, Zeng Z (2012b) Windows QTL Cartographer 2.5. Department of Statistics, North Carolina State University, Raleigh, NC. http://statgen.ncsu.edu/qtlcart/WQTLCart.htm

[CR39] Wang S, Wu K, Yuan Q, Liu X, Liu Z, Lin X, Zeng R, Zhu H, Dong G, Qian Q, Zhang G, Fu X (2012). Control of grain size, shape and quality by *OsSPL16* in rice. Nat Genet.

[CR40] Wei X, Qiu J, Yong K, Fan J, Zhang Q, Hua H, Liu J, Wang Q, Olsen KM, Han B, Huang X (2021). A quantitative genomics map of rice provides genetic insights and guides breeding. Nat Genet.

[CR41] Wei X, Xu J, Guo H, Jiang L, Chen S, Yu C, Zhou Z, Hu P, Zhai H, Wan J (2010). *DTH8* suppresses flowering in rice, influencing plant height and yield potential simultaneously. Plant Physiol.

[CR42] Xi ZY, He FH, Zeng RZ, Zhang ZM, Ding XH, Li WT, Zhang GQ (2006). Development of a wide population of chromosome single-segment substitution lines in the genetic background of an elite cultivar of rice (Oryza sativa L). Genome.

[CR43] Xu J, Zhao Q, Du P, Xu C, Wang B, Feng Q, Liu Q, Tang S, Gu M, Han B, Liang G (2010). Developing high throughput genotyped chromosome segment substitution lines based on population whole-genome re-sequencing in rice (Oryza sativa L.). BMC Genom.

[CR44] Xue W, Xing Y, Weng X, Zhao Y, Tang W, Wang L, Zhou H, Yu S, Xu C, Li X, Zhang Q (2008). Natural variation in *Ghd7* is an important regulator of heading date and yield potential in rice. Nat Genet.

[CR45] Yan W, Liu H, Zhou X, Li Q, Zhang J, Lu L, Liu T, Liu H, Zhang C, Zhang Z, Shen G, Yao W, Chen H, Yu S, Xie W, Xing Y (2013). Natural variation in Ghd71 plays an important role in grain yield and adaptation in rice. Cell Res.

[CR46] Yan WH, Wang P, Chen HX, Zhou HJ, Li QP, Wang CR, Ding ZH, Zhang YS, Yu SB, Xing YZ, Zhang QF (2011). A major QTL, *Ghd8*, plays pleiotropic roles in regulating grain productivity, plant height, and heading date in rice. Mol Plant.

[CR47] Yano M, Harushima Y, Nagamura Y, Kurata N, Minobe Y, Sasaki T (1997). Identification of quantitative trait loci controlling heading date in rice using a high-density linkage map. Theor Appl Genet.

[CR48] Yano M, Katayose Y, Ashikari M, Yamanouchi U, Monna L, Fuse T, Baba T, Yamamoto K, Umehara Y, Nagamura Y, Sasaki T (2000). *Hd1*, a major photoperiod sensitivity quantitative trait locus in rice, is closely related to the *Arabidopsis* flowering time gene *CONSTANS*. Plant Cell.

[CR49] Yu B, Lin Z, Li H, Li X, Li J, Wang Y, Zhang X, Zhu Z, Zhai W, Wang X, Xie D, Sun C (2007). *TAC1*, a major quantitative trait locus controlling tiller angle in rice. Plant J.

[CR50] Yuan R, Zhao N, Usman B, Luo L, Liao S, Qin Y, Nawaz G, Li R (2020). Development of chromosome segment substitution lines (CSSLs) derived from guangxi wild rice (Oryza rufipogon Griff) under rice (Oryza sativa L) background and the identification of QTLS for plant architecture, agronomic traits and cold tolerance. Genes.

[CR51] Zamir D (2001). Improving plant breeding with exotic genetic libraries. Nat Rev Genet.

[CR52] Zeng D, Tian Z, Rao Y, Dong G, Yang Y, Huang L, Leng Y, Xu J, Sun C, Zhang G, Hu J, Zhu L, Gao Z, Hu X, Guo L, Xiong G, Wang Y, Li J, Qian Q (2017). Rational design of high-yield and superior-quality rice. Nature Plants.

[CR53] Zhang B, Shang L, Ruan B, Zhang A, Yang S, Jiang H, Liu C, Hong K, Lin H, Gao Z, Hu J, Zeng D, Guo L, Qian Q (2019). Development of three sets of high-throughput genotyped rice chromosome segment substitution lines and qtl mapping for eleven traits. Rice.

[CR54] Zhang G, Zeng RZ, Zhang Z, Ding XH, Li WT, Liu GM, He F-H, Tulukdar A, Huang CF, Xi ZY, Qin LJ, Shi JQ, Zhao FM, Feng MJ, Shan ZL, Chen L, Guo XQ, Zhu HT, Lu YG (2004). The construction of a library of single segment substitution lines in rice (*Oryza sativa* L.). Rice Genet Newsl.

[CR55] Zhang Q (2007). Strategies for developing green super rice. Proc Natl Acad Sci USA.

[CR56] Zhang Y, Luo L, Xu C, Zhang Q, Xing Y (2006). Quantitative trait loci for panicle size, heading date and plant height co-segregating in trait-performance derived near-isogenic lines of rice (Oryza sativa). Theor Appl Genet.

[CR57] Zhao J, Chen H, Ren D, Tang H, Qiu R, Feng J, Long Y, Niu B, Chen D, Zhong T, Liu YG, Guo J (2015). Genetic interactions between diverged alleles of *Early heading date 1* (*Ehd1*) and *Heading date 3a* (*Hd3a*)/ *RICE FLOWERING LOCUS T1* (*RFT1*) control differential heading and contribute to regional adaptation in rice (*Oryza sativa*). New Phytol.

[CR58] Zhao Q, Huang X, Lin Z, Han B (2010). SEG-map: a novel software for genotype calling and genetic map construction from next-generation sequencing. Rice.

[CR59] Zhu J, Niu Y, Tao Y, Wang J, Jian J, Tai S, Li J, Yang J, Zhong W, Zhou Y, Liang G (2015). Construction of high-throughput genotyped chromosome segment substitution lines in rice (Oryza sativa L.) and QTL mapping for heading date. Plant Breed.

[CR60] Zhu W, Lin J, Yang D, Zhao L, Zhang Y, Zhu Z, Chen T, Wang C (2009). Development of chromosome segment substitution lines derived from backcross between two sequenced rice cultivars, Indica recipient 93–11 and Japonica donor nipponbare. Plant Mol Biol Report.

