# Supplementary figures and images for: Whole-Genome Sequencing of 117 Chromosome Segment Substitution Lines for Genetic Analyses of Complex Traits in Rice
Source: Rice (N Y). 2022 Jan 13;15:5. doi: 10.1186/s12284-022-00550-y (PMC8758858; doi:10.1186/s12284-022-00550-y)

Chr1 Chr2 Chr3 Chr4 Chr5 Chr6 Chr7 Chr8 Chr9 Chr10 Chr11 Chr12

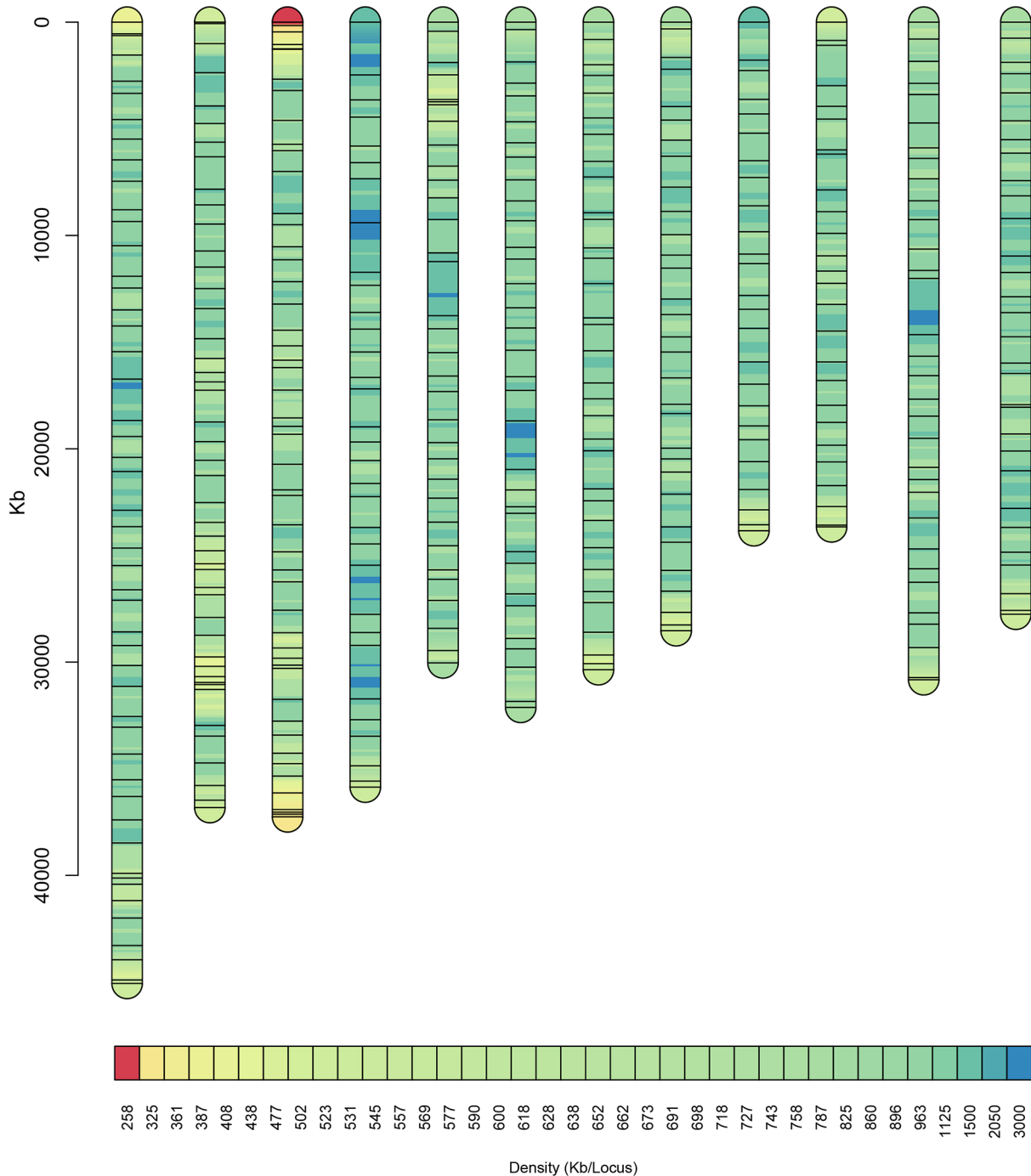

Supplement: Supplementary file 1 — Additional file 1. Fig. S1 Location of 396 InDel marker primers on chromosomes; the black bar represents a marker. [file 12284_2022_550_MOESM1_ESM.pdf]

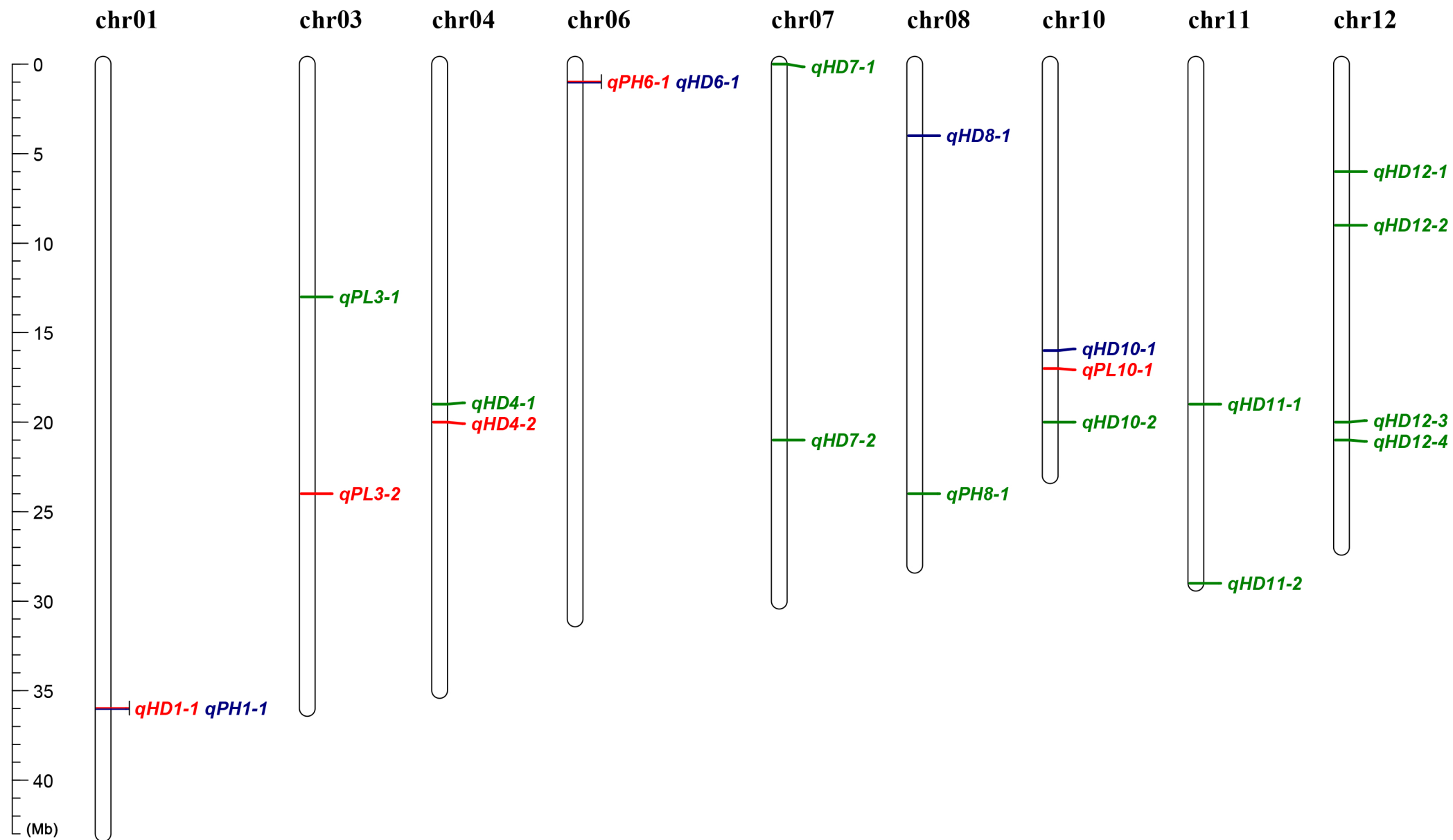

Supplement: Supplementary file 4 — Additional file 4. Fig. S2 QTL distribution on chromosomes. The blue font denotes loci found in both environments, the red font denotes loci that can only be found in Shanghai, and the green font denotes loci that can only be found in Hangzhou. [file 12284_2022_550_MOESM4_ESM.pdf]

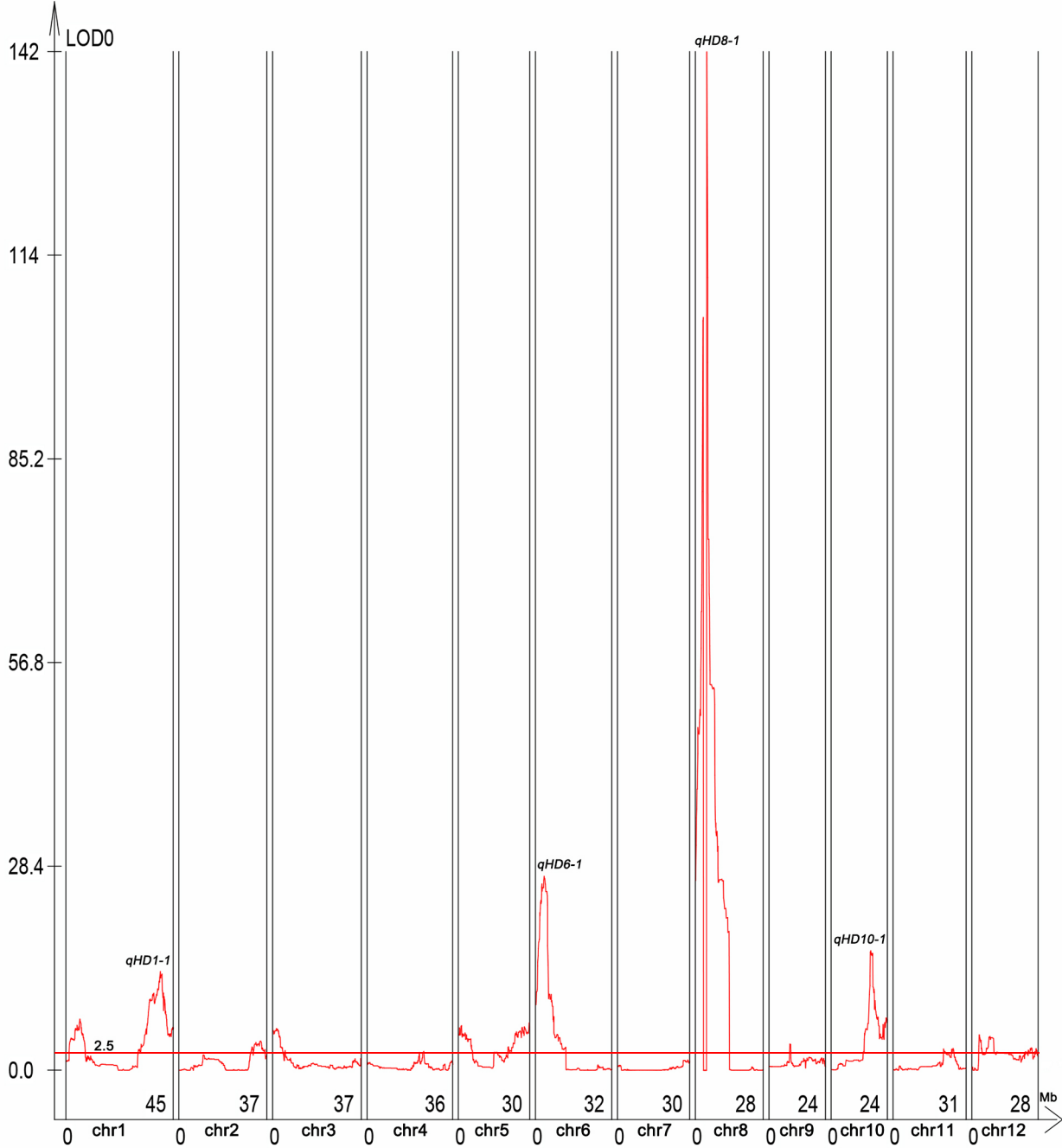

Supplement: Supplementary file 5 — Additional file 5. Fig. S3 Mapping of heading date in the BC3F1 population by WinQTLCart. [file 12284_2022_550_MOESM5_ESM.pdf]

*Hd3a* promoter region

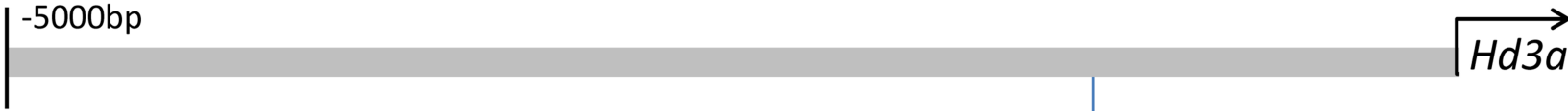

| Position | 1308 |
|----------|------|
| HHZ      | T    |
| BAS      | -    |

Supplement: Supplementary file 6 — Additional file 6. Fig. S4 Nucleotide polymorphism of the Hd3a promoter between HHZ and BAS. [file 12284_2022_550_MOESM6_ESM.pdf]
