# Supplementary material for: Whole-Genome Sequencing of 117 Chromosome Segment Substitution Lines for Genetic Analyses of Complex Traits in Rice
Source: Rice (N Y). 2022 Jan 13;15:5. doi: 10.1186/s12284-022-00550-y (PMC8758858; doi:10.1186/s12284-022-00550-y)
Supplement: Supplementary file 2 — Additional file 2. Table S1 Distribution of InDel markers on 12 rice chromosomes used to construct the set of CSSLs. [file 12284_2022_550_MOESM2_ESM.docx]

**Additional file 2: Table S1** Distribution of InDel markers on 12 rice chromosomes used to construct the set of CSSLs

| Chromosome | No. of InDel makes | Chromosome length (Mb) | Destiny (Mb) |
| --- | --- | --- | --- |
| 1 | 47 | 43.2 | 0.92 |
| 2 | 44 | 35.8 | 0.81 |
| 3 | 48 | 36.3 | 0.76 |
| 4 | 30 | 35.4 | 1.18 |
| 5 | 31 | 29.9 | 0.96 |
| 6 | 31 | 31.1 | 1.00 |
| 7 | 31 | 29.6 | 0.95 |
| 8 | 29 | 28.3 | 0.98 |
| 9 | 23 | 22.9 | 1.00 |
| 10 | 24 | 23.1 | 0.96 |
| 11 | 30 | 28.9 | 0.96 |
| 12 | 28 | 27.4 | 0.98 |
| total | 396 | 371.9 | 0.94 |
