# Supplementary material for: Whole-Genome Sequencing of 117 Chromosome Segment Substitution Lines for Genetic Analyses of Complex Traits in Rice
Source: Rice (N Y). 2022 Jan 13;15:5. doi: 10.1186/s12284-022-00550-y (PMC8758858; doi:10.1186/s12284-022-00550-y)
Supplement: Supplementary file 7 — Additional file 7. Table S3 Primers for yeast two-hybrid vectors. [file 12284_2022_550_MOESM7_ESM.docx]

**Additional file 7: Table S3** Primers for yeast two-hybrid vectors

| **Name** | **Sequence (5'-3')** |
| --- | --- |
| RFT1-AD-F | ATGGAGGCCAGTGAATTCATGGCCGGCAGCGGCAGGGACGATC |
| RFT1-AD-R | AGCTCGAGCTCGATGGATCCCCTAGGGGTAGACCCTCCTGC |
| GF14a-BD-F | GCCATGGAGGCCGAATTCATGGCGGCGGCGGCGGGAGGAG |
| GF14a-BD-R | CTGCAGGTCGACGGATCCCTAGTGCTCATCCTCAGGCTTGG |
| GF14b-BD-F | GCCATGGAGGCCGAATTCATGTCGGCACAGGCGGAGCTTTC |
| GF14b-BD-R | CTGCAGGTCGACGGATCCTTACTGCCCCTCGCTGGAGTCGC |
| GF14c-BD-F | GCCATGGAGGCCGAATTCATGTCTCGGGAGGAGAATGT |
| GF14c-BD-R | CTGCAGGTCGACGGATCCCTTACTGGCCCTCGCAGGCGT |
| GF14d-BD-F | GCCATGGAGGCCGAATTCATGTCGCCGGCGGAGCCGACGAG |
| GF14d-BD-R | CTGCAGGTCGACGGATCCTCACTGATCCCCAGGCTCTTTTG |
| GF14e-BD-F | GCCATGGAGGCCGAATTCATGTCGCAGCCTGCTGAGCTTTC |
| GF14e-BD-R | CTGCAGGTCGACGGATCCTCACTGTCCATCTCCTGATTCGC |
| GF14f-BD-F | GCCATGGAGGCCGAATTCATGTCGCCTGCTGAGGCATCGCG |
| GF14f-BD-R | CTGCAGGTCGACGGATCCTTAGTGGCCCTCTCCTTCAGGCT |
| T7-sequencing-primer | TAATACGACTCACTATAGGGC |
| 3’AD-sequencing-primer | AGATGGTGCACGATGCACAG |
| 3’BD-sequencing-primer | TTTTCGTTTTAAAACCTAAGAGTC |
